# Selection and Validation of 48 KASP Markers for Variety Identification and Breeding Guidance in Conventional and Hybrid Rice (*Oryza sativa* L.)

**DOI:** 10.1186/s12284-022-00594-0

**Published:** 2022-09-24

**Authors:** Weijie Tang, Jing Lin, Yanping Wang, Hongzhou An, Haiyuan Chen, Gen Pan, Suobing Zhang, Baowei Guo, Kun Yu, Huayong Li, Xianwen Fang, Yunhui Zhang

**Affiliations:** 1 Provincial Key Laboratory of Agrobiology, Institute of Germplasm Resources and Biotechnology, Jiangsu Academy of Agricultural Sciences, Nanjing, People’s Republic of China; 2 Jiangsu Co-Innovation Center for Modern Production Technology of Grain Crops, Yangzhou University, Yangzhou, People’s Republic of China; 3 Jiangsu Key Laboratory of Crop Genetics and Physiology, Agricultural College of Yangzhou University, Yangzhou, People’s Republic of China; 4 The Key Laboratory of Crop Genetics and Breeding of Hebei Province, Institute of Cereal and Oil Crops, Hebei Academy of Agriculture and Forestry Sciences, Shijiazhuang, People’s Republic of China; 5 Institute of Bast Fiber Crops, Chinese Academy of Agricultural Sciences, Changsha, People’s Republic of China

**Keywords:** KASP, Rice, Variety identification, Marker-assisted breeding

## Abstract

**Background:**

Breeding of conventional and hybrid rice (*Oryza sativa* L.) have solved hunger problems and increased farmers' income in the world. Molecular markers have been widely used in marker-assisted breeding and identification of larger numbers of different bred varieties in the past decades. The recently developed SNP markers are applied for more stable and detectable compared with other markers. But the cost of genotyping lots SNPs is high. So, it is essential to select less representative SNPs and inexpensive detecting methods to lower the cost and accelerate variety identification and breeding process. KASP (Kompetitive Allele-Specific PCR) is a flexible method to detect the SNPs, and large number of KASP markers have been widely used in variety identification and breeding. However, the ability of less KASP markers on massive variety identification and breeding remains unknown.

**Results:**

Here, 48 KASP markers were selected from 378 markers to classify and analyze 518 varieties including conventional and hybrid rice. Through analyzing the population structure, the 48 markers could almost represent the 378 markers. In terms of variety identification, the 48 KASP markers had a 100% discrimination rate in 53 conventional *indica* varieties and 193 hybrid varieties, while they could distinguish 89.1% conventional *japonica* rice from different breeding institutes. Two more markers added would increase the ratio from 68.38 to 77.94%. Additionally, the 48 markers could be used for classification of subpopulations in the bred variety. Also, 8 markers had almost completely different genotypes between *japonica* and *indica*, and 3 markers were found to be very important for *japonica* hybrid rice. In hybrid varieties, the heterozygosity of chromosomes 3, 6 and 11 was relatively higher than others.

**Conclusions:**

Our results showed that 48 KASP markers could be used to identify rice varieties, and the panel we tested could provide a database for breeders to identify new breeding lines. Also, the specific markers we found were useful for marker-assisted breeding in rice, including conventional and hybrid.

**Supplementary Information:**

The online version contains supplementary material available at 10.1186/s12284-022-00594-0.

## Background

Rice is a vital crop that feeds almost half the world’s population. In the past several decades, there has been great progress in rice variety identification and breeding. A few bred varieties, such as the ‘miracle rice’ IR8, solved the lodging problem and increased production (Peng et al. 2010).

With the progress of rice breeding, there are increasingly more bred varieties. Distinguishing a new variety from others is a problem that needs to be resolved quickly. In the past, researchers used the distinctness, uniformity, and stability (DUS) tests to identify these varieties (Aravind et al. 2019). Using this test, researchers need to record a lot of phenotype data from the seedling to maturing stage. Subsequently, the data must be inputted to the server and analyzed. There are several weak points in this approach, such as data instability in different years, time consumption and labor intensity of the process.

To overcome these disadvantages, molecular markers have been explored to assist in identifying varieties in recent years, such as 12 microsatellite markers for authentication in rice (Bonow et al. 2009). Recently, microsatellite markers have been replaced by single nucleotide polymorphism (SNP) markers due to their stability and easy detectability. Many genotyping methods have emerged, such as microarray (Lou et al. 2015; Lu et al. 2017), genotyping by sequencing (GBS) (An et al. 2019; Lin et al. 2020b; Tang et al. 2016), high resolution melting (HRM) (Wang et al. 2013), and kompetitive allele-specific PCR (KASP) (Shen et al. 2021; Steele et al. 2020).

Compared with other genotyping method, KASP was more popular in varietal identification recently. The conventional simple satellite repeat (SSR) or insertion/deletions (InDels) markers had been proved that they could be useful for variety identification and estimation of subpopulation (Rahman et al. 2012; Kumar et al. 2019; Castellana et al. 2020), but the cost of KASP markers which was also used for identification and estimation was lower (Shikari et al. 2021). Additionally, the SSR markers were not evenly distributed in the genome and the genotyping was tedious and time consuming (Semagn et al. 2014). Whole-genome sequencing may be the best way to identify different varieties and have highest accuracy in identification, but the cost of library construction was higher compared with other methods. GBS had a lower cost in library construction, but the distribution of markers are not evenly as other method (Huang and Han 2014). Microarray was a better choice to make the markers distribute evenly, but it was not cheap and not flexible in varietal identification (Semagn et al. 2014). So, the above three methods were not suitable for massive varietal identification perfectly. As previous study showed, KASP was a better choice with more flexibility and higher efficiency (Rasheed et al. 2017). We could add more markers for the polymorphic detection in a specific population according to some newly identified functional genes. Overall, KASP or less representative KASP markers was more suitable for varietal identification compared with other markers in consideration of labor, time, cost, distribution and flexibility.

Additionally, it has been widely used for genotyping in crops, especially in rice. KASP was suitable for identifying the varieties in specific subpopulations of rice, such as *Basmati* and had more accurate identification rate than SSR markers (Steele et al. 2020). In Korea *japonica*, the number of SSR markers was limited for the construction of genetic maps and quantitative trait locus (QTL) mapping studies because of the polymorphisms. The researchers developed KASP markers from the resequencing data and 205 KASP markers were used for constructing genetic map (Cheon et al. 2018). Furthermore, KASP could be used for rice specific gene genotyping, such as *Wx*, *ALK*, and *BADH2 *which had much influence in the quality of rice, and has been applied to improve rice quality through marker-assisted selection (MAS). The high-throughput of KASP could help accelerating the process of rice breeding lines selection (Addison et al. 2020; Yang et al. 2019a).In *indica* breeding, the researchers used the resequencing data to generate 1.3 million potential KASP assay designs and 377,178 polymorphic KASP design sites per cross averagely. These KASP markers which could replace SSR makers were useful to whole-genome *indica* breeding (Steele et al. 2018).In another study, a core SNP arrays consisting of 467 KASP markers were proved to be used in germplasm assessment, genetic diversity and population evaluation in rice (Yang et al. 2019b). From the above study, we could see that KASP was widely used for varietal identification, genetic map construction, MAS, genetic diversity, population evaluation and breeding in rice.

Although large numbers of KASP markers could be applied for genotyping in rice identifying and molecular breeding, the cost will be high if hundreds of thousands of varieties need to be analyzed. So, it is essential to develop less representative KASP markers for the breeders to lower the cost. It is still unknown whether less KASP markers could work in identifying rice varieties and assisting breeding, including conventional and hybrid rice, especially in China. Therefore, we used more than 500 varieties, including conventional and hybrid rice, to validate the 48 KASP markers selected from 378 KASP markers for variety identification and found some markers useful for rice breeding.

To determine the relationship of less KASP markers and variety identification, we herein used 378 markers to test the effect of KASP markers with a population consisting of *japonica*, *indica*, and *aus*. Subsequently, we selected 48 markers to verify the effect using 518 bred varieties, including conventional and hybrid rice, and found that the markers could distinguish 83.40% of the varieties. Particularly, the discrimination rate was 100% in conventional *indica* and hybrid varieties. Meanwhile, we found some specific markers that played a vital role in subgroup differences and heterosis formation. Therefore, the 48 KASP markers could also be used for marker-assisted breeding.

## Main text

### KASP Markers Were Developed Using a Diverse Rice Population from Asia

**Fig. 1:**
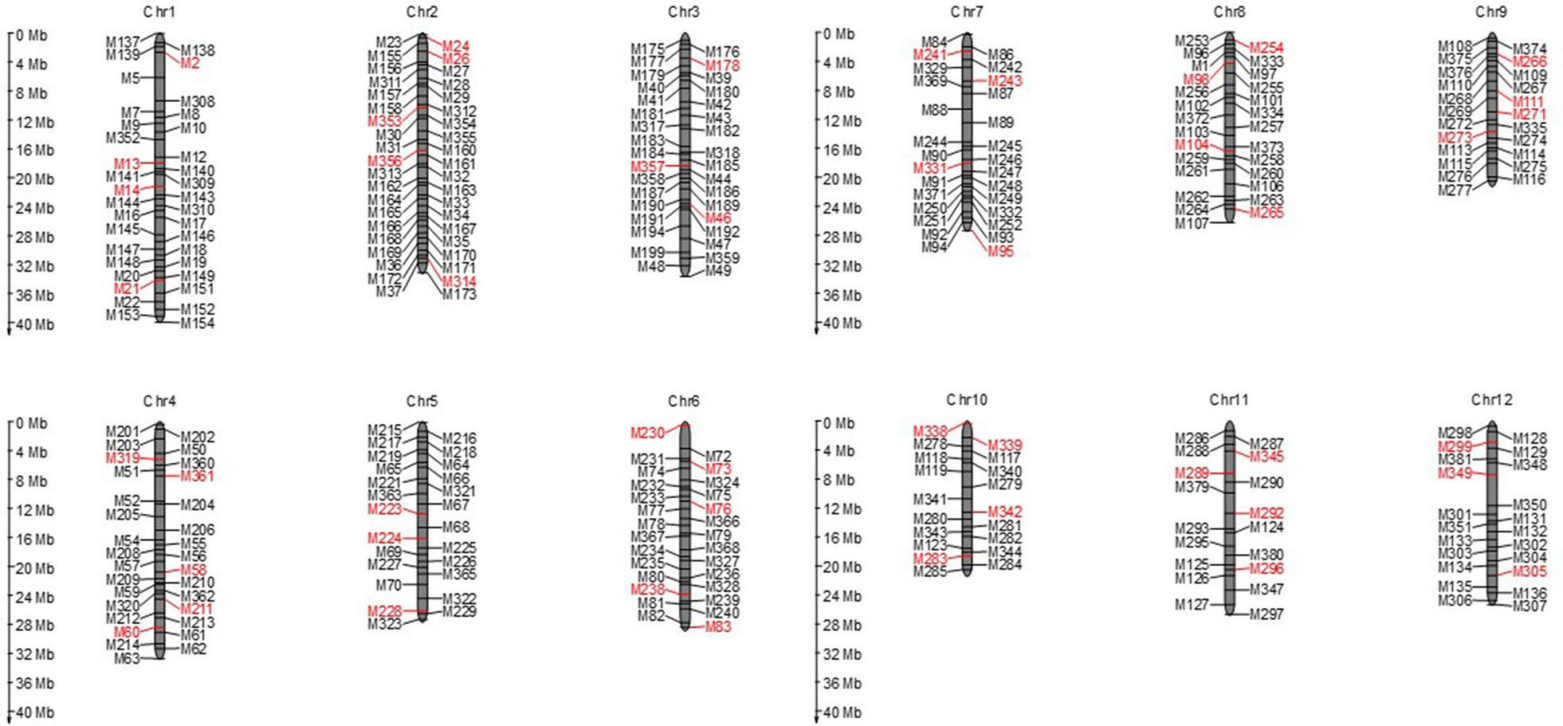
Distribution of filtered 331 markers and selected 48 markers (red) on 12 chromosomes

**Fig. 2:**
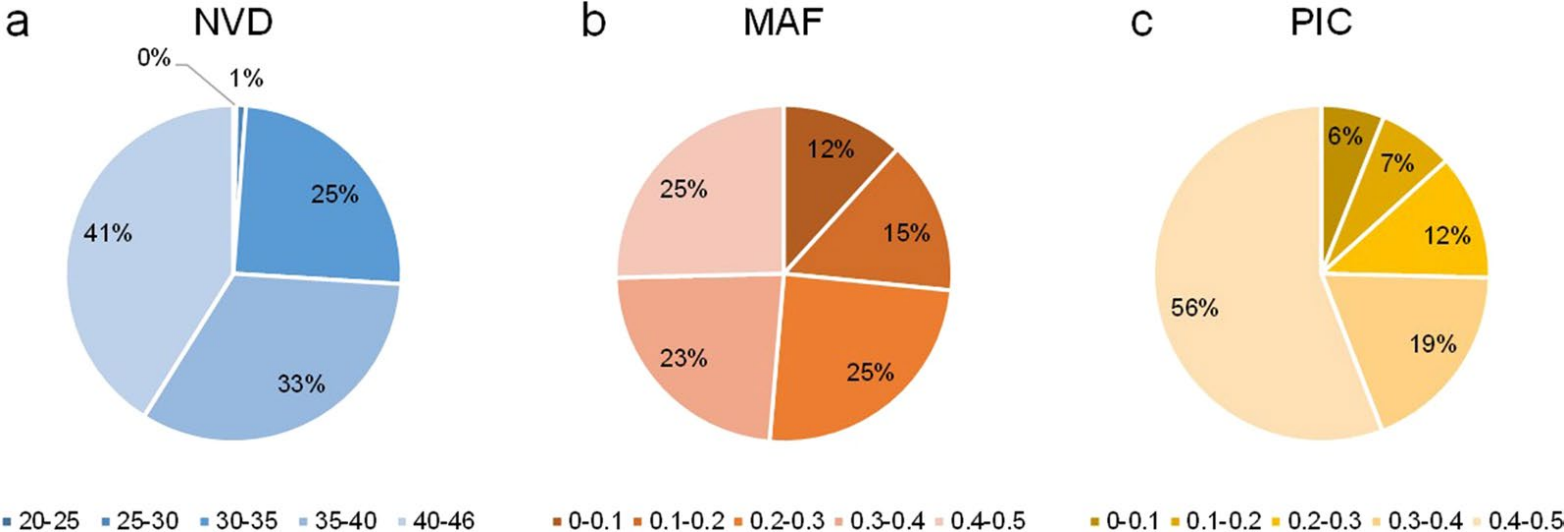
The data of number of varieties detected (NVD) (**a**), MAF (**b**) and PIC (**c**) of 331markers

To determine whether the 378 KASP markers (Additional file 1: Table S1) were effective, we used the KASP markers to genotype a diverse rice population. The population consisted of *japonica* (20), *indica* (21), and *aus* (5) subpopulations, and the varieties came from east (33), southeast (8), and south Asia (5) (Additional file 2: Table S2). After filtering with polymorphism, we identified 331 KASP markers that were effective (Additional file 3: Table S3). These markers were well distributed across all 12 chromosomes (Fig. 1). The distance between markers ranged from 0.41 to 4.64 Mb, and the average distance was 1.13 Mb. Among all markers, 98.8% showed missing genotyping less than 16 varieties (Fig. 2a), and the minor allele frequency (MAF) of most markers was more than 0.2 (Fig. 2b). The polymorphism information content (PIC) of 74.6% markers was larger than 0.3 (Fig. 2c). These results provided the basis for subsequent variety identification and finding the specific markers for breeding.

### Markers Selected and Used for Variety Identification

**Fig. 3:**
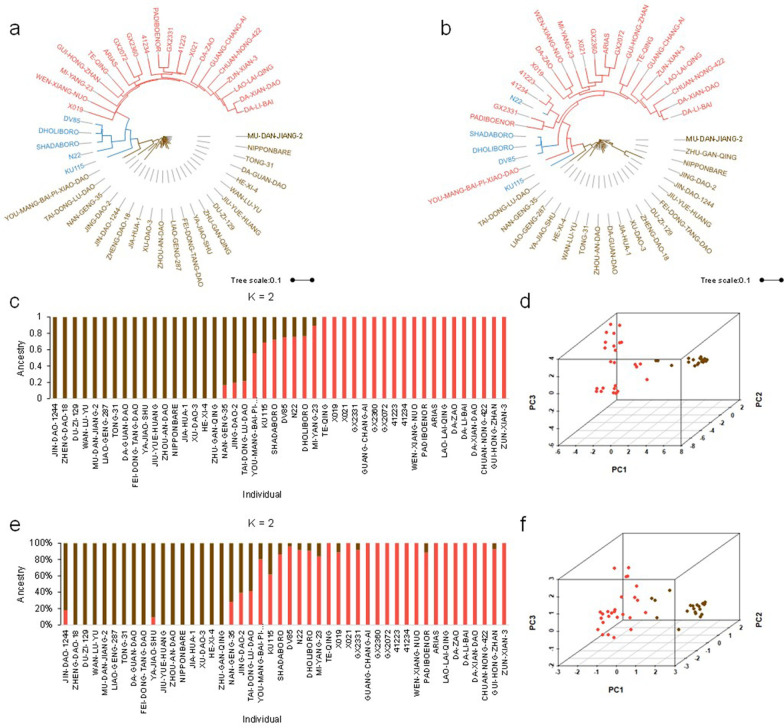
Analysis of the 46 various varieties using KASP markers. **a**, **c**, **d** The phylogenetic tree, population structure and PCA of the test population using 331 KASP markers. **b**, **e**, **f** The phylogenetic tree, population structure and PCA of the test population using 48 KASP markers. The brown indicated the *japonica* varieties while light red and blue represented the *indica* and *aus* varieties in a, b. The brown indicated the subpopulation 1 while light red represented subpopulation 2 in **d**, **f**

KASP was an efficient tool to distinguish various varieties depending on the number of markers used; thus, we sorted the markers based on the detection ratio (> 0.8), MAF > 0.2 and PIC > 0.3. As a result, 171 markers remained, from which we selected 48 markers according to the physical position of each marker (Fig. 1, Additional file 4: Table S4). To verify the representativeness of the 48 markers selected, the phylogenetic tree was constructed and the tree both revealed two distinct groups, with subpopulation 1 into one group, and the other was subpopulation 2 (Fig. 3a, b). Secondly, we analyzed the population structure with 331 markers and 48 markers for each of the 46 varieties. The structure analysis was performed by setting the range of K as 1 to 9. The minimum value of CV error in both analyses was when K was 2 (Fig. 3c, e, Additional file 5: Table S5). At last, two groups were separated by principal component analysis corresponding to subpopulation 1 and subpopulation 2 (Fig. 3d, f, Additional file 6: Table S6). The above results showed that 48 markers could almost represent the 331 markers.

After detection of the 518 varieties (193 hybrid varieties and 325 conventional varieties) (Additional file 7: Table S7), we found that all markers had polymorphisms (Additional file 8: Table S8 and Additional file 9: Table S9). The SNP detection rate was 91.16%. We focused on the resolution of the markers in the panel and found that the markers we selected differed in 83.40% of all varieties (432/518). Then, we separated the whole population into subpopulations, namely *japonica* (conventional and hybrid) and *indica* (conventional and hybrid), to observe the duplications of SNP combinations (the same combination among all varieties was defined the duplications). As a result,100% of conventional *indica* varieties (53/53), 100% of hybrid varieties (193/193), and 68.38% of conventional *japonica* varieties (186/272) had unique SNP combinations (Table 1).

**Table 1:** Summary of the detection rate, heterozygous rate, and identification rate using 48 and 50 markers

Population	Detection rate (%)	Average heterozygous rate	Identifying rate^a^
Hybrid variety	91.98	41.73%	100%
*Indica* (175)	97.57	40.62%	100%
*Japonica* (18)	91.40	51.84%	100%
Conventional variety	92.69	NA	73.54% (81.54%)
*Indica* (53)	85.26	NA	100%
*Japonica* (272)	94.13	NA	68.38% (77.94%)

^a^Identifying rate of 48 markers (identifying rate of 50 markers)

### Analysis of 48 Markers in Conventional Varieties

In conventional varieties, the SNP detection rate was 92.69% (Table 1). There were 29.78% duplications in conventional *japonica* and 0% in conventional *indica* or hybrid varieties, indicating that the *japonica* varieties had more similarities than others, and we should add more markers to assess them. To decrease the ratio of varieties that could not be distinguished, we added two markers (M54, M234) according to their physical position. After that, the identifying ratio increased from 68.38 to 77.94% (Table 1). It was demonstrated that more markers could be added as expected and a higher ratio would be obtained. Additionally, after analyzing the conventional *japonica* varieties that could not be discriminated from each other using the 48 KASP markers, we found that almost all the varieties came from the same breeding institutes, and the varieties may share the same parents. After deleting the possible redundant sib lines from the same breeding institutes (Additional file 10: Table S10), the identifying rate in conventional *japonica* of 48 KASP markers could be 89.1% (106/119). The above results showed that the 48 or 50 markers could be very useful for identification of conventional varieties.

**Fig. 4:**
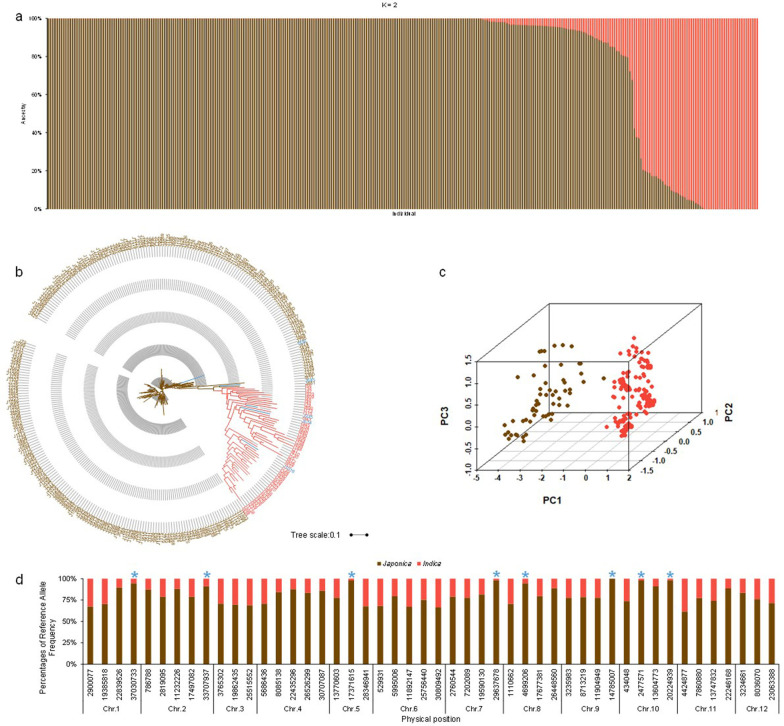
Analysis of the 48 KASP markers in conventional varieties. **a**–**c** The phylogenetic tree, population structure and PCA of the conventional varieties using 48 KASP markers. **d** The difference of reference allele frequency between *japonica* and *indica*. The blue star indicated the sites which showed almost completely different alleles. The brown indicated the *japonica* varieties while light red represented the *indica* varieties in **b**. The blue indicated that the varieties with the classification of the phylogenetic tree different from the original annotation coming from the breeding institutes in **b**. The brown indicated the subpopulation 1 while light red represented subpopulation 2 in **c**

To verify the 48 markers could be used for population analysis and breeding of rice conventional varieties, we analyzed the population structure and constructed the phylogenetic tree. The K which had minimum CV error showed that the population could divided into two subspecies, subpopulation 1 and subpopulation 2 (Fig. 4a, Additional file 11: Table S11). Also, the result of phylogenetic tree was the same as that of population structure analysis (Fig. 4b). At last, the result of PCA again verified the above two results (Fig. 4c, Additional file 12: Table S12). From the results, we approved that the 48 markers were suitable for rice conventional varieties analysis, and the breeders could use the 48 KASP markers and the panel to identify the breeding lines.

To detect the genetic differences between subgroups and identify if some KASP markers could be used for breeding, we summarized the ratio of reference allele frequency and found that eight markers (M21, M314, M224, M95, M98, M273, M342, M283) in conventional rice were almost completely different between *indica* (< 10%) and *japonica* rice (> 90%) (Fig. 4d). To find the highest percentage of reference allele frequencies of markers between *japonica* and *indica*, we conculcated the percentage of each marker and average percentage of each chromosome (Additional file 13: Table S13). The result showed that almost 75.00% (36/48) markers had more than 95% reference allele frequency and only two markers (M345, M349) had less than 80% reference allele frequency in *japonica*. Conversely, 33.33% (16/48) markers had more than 30% reference allele frequency and no markers exceeded half of reference allele frequency in *indica* (Additional file 13: Table S13). Also, we found that the chromosome 3 was the least and 10 was the highest in average percentages of reference allele frequency (Fig. 4d). From the above result, we could see the *indica* subpopulation had more diversity and these KASP markers could be used for assisting breeders to determinate the subgroup of varieties.

### Analysis of 48 Markers in Hybrid Varieties

In comparison, in hybrid varieties, the SNP detection rate was 91.98%, and the heterozygous rate was 41.73% (Table 1). We used 48 markers to assess the difference between them and found that each hybrid variety had unique combinations. This showed that hybrid varieties had more polymorphisms than conventional varieties, especially in *japonica* hybrids. Additionally, we found that the heterozygous rate of each variety in *japonica* hybrids was 35.42–67.39%, and the average rate was 51.84%, while it was 12.77–60.42% and 40.62% in *indica* hybrids (Table 1). This indicated that *japonica* hybrids may require a higher heterozygous rate and could show stronger heterosis.

To study the ability of the 48 KASP markers for characteristic analysis in hybrid varieties, we analyzed the population structure. As results, the ΔK value was much higher for the model parameter K = 2 than other values of K (Additional file 14: Table S14). The 193 accessions could be divided into two subpopulations. The result of phylogenetic tree construction and PCA showed the same with that of population structure analysis (Additional file 15: Table S15). The result showed that the 48 KASP markers could be used for classification of subpopulations in hybrid varieties.

**Fig. 5:**
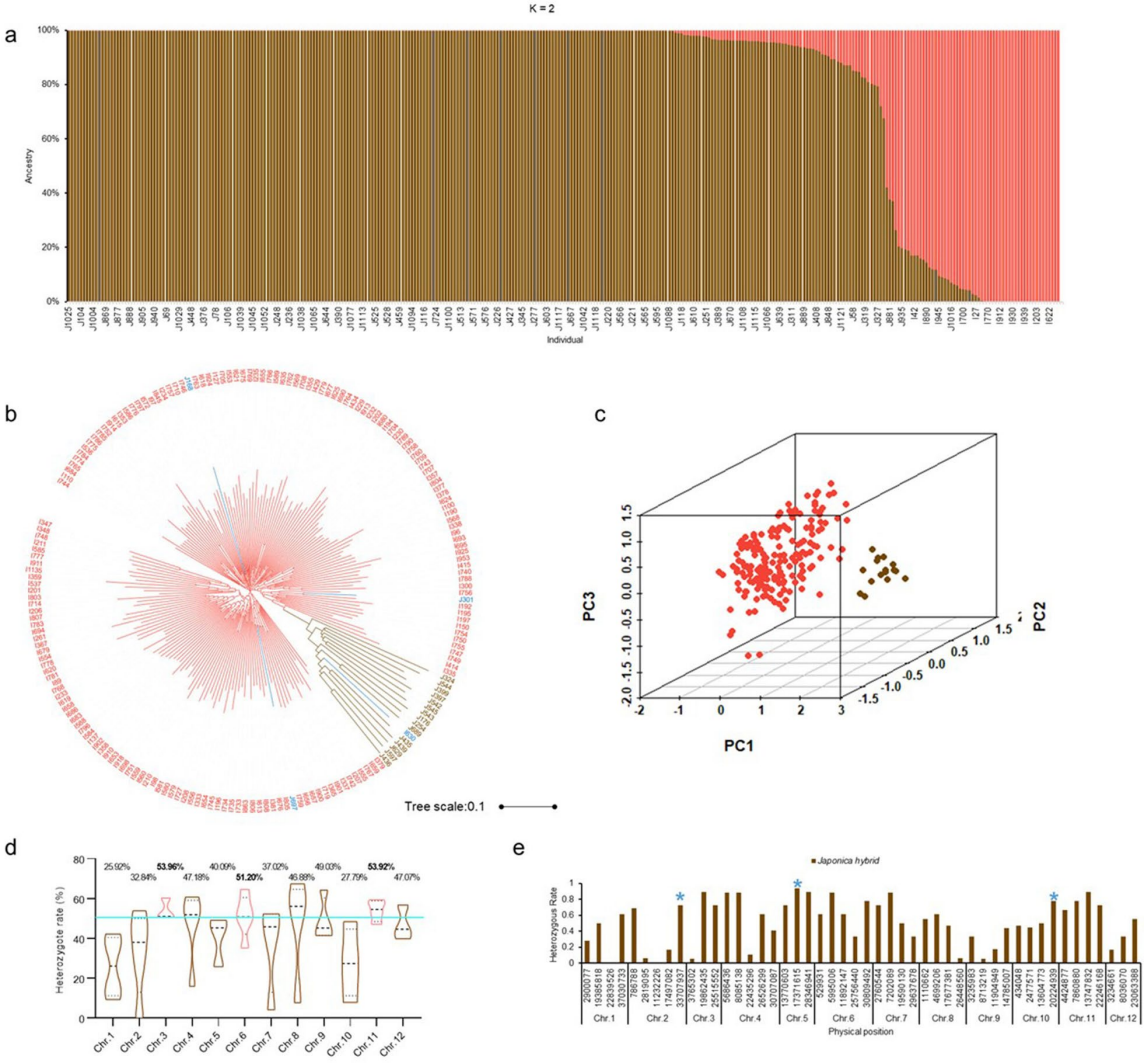
Analysis of the 48 KASP markers in hybrid varieties. **a**–**c** The phylogenetic tree, population structure and PCA of the hybrid varieties using 48 KASP markers. **d** The heterozygous rate of each chromosome. **e** The heterozygous rate of japonica hybrid variety. The blue star indicated three sites with higher heterozygous rate. The brown indicated the *japonica* varieties while light red represented the *indica* varieties in **b**. The blue indicated that the varieties with the classification of the phylogenetic tree different from the original annotation coming from the breeding institutes in **b**. The brown indicated the subpopulation 1while light red represented subpopulation 2 in **c**

To observe the heterozygous rate of each chromosome, we summarized the heterozygous rate. In all 12 chromosomes, 3, 6 and 11 had more heterozygosity than the others (Fig. 5d). This indicated that the 3 chromosomes may have more genes that contributed to heterosis-related traits, such as yield and heading date. Through a literature review, we observed that some genes were located on the 3 chromosomes, such as *GS3*, *Ha3a*, *MOC1* and *IPA1* (Jiao et al. 2010; Lin et al. 2020a; Mao et al. 2010; Tamaki et al. 2007).

To determine the difference between *japonica* and *indica* hybrid varieties, we calculated the heterozygous rate of each site and found that two markers (M21, M283) had a higher percentage in *japonica* than *indica* hybrid varieties (Table 2). Conversely, four markers (M26, M178, M265, M111) predominated in the *indica* hybrid (Table 2). Thus, the KASP markers could be used for detecting which subpopulation the variety belonged to, and the markers would help rice breeders to identify the pedigree earlier. More importantly, we found that three markers (M224, M283, M314) which had higher heterozygous rate (> 0.7) in *japonica* hybrids shared the same positions as the markers which had almost completely different genotypes between *japonica* and *indica* conventional varieties (Fig. 5e). This indicated that the *japonica* hybrids rice showed heterosis need these differentiated sites between *japonica* and *indica* more. Therefore, the identified markers should be helpful for rice breeders to select *japonica* hybrids in rice breeding.

**Table 2:** Heterozygous rate of markers showed differences between *japonica* and *indica*

Marker ID	Chr	Position	Allele	Heterozygous rate	
				*Japonica* (%)	*Indica* (%)
M21	1	37,030,733	A/G	61.11	4.02
M283	10	20,224,939	A/G	77.78	0.58
M26	2	2,819,095	A/G	6.25	58.29
M178	3	3,765,302	G/A	5.88	54.91
M265	8	26,448,560	C/A	6.25	62.83
M111	9	8,713,219	A/G	5.56	52.87

## Discussion

### KASP Was a Better Selection for Identifying Varieties

Compared with SSR markers, the KASP had advantages with a higher efficiency and labor-saving (Rasheed et al. 2017). The SNPs produced by GBS were mostly used for population structure analysis (Pereira-Dias et al. 2019). Whole-genome sequencing was frequently used for scanning the polymorphism of varieties and the polymorphisms were used for GWAS in rice (Chen et al. 2014; Crowell et al. 2016; Yano et al. 2016). KASP markers had been used for varietal identification (Swisher Grimm and Porter 2020; Wang et al. 2021; Steele et al. 2020). In our study, we selected and validated less KASP markers (about one eighth of all 378 markers) for identifying varieties, and the cost decreased much compared with the 378 markers correspondingly. So, the selected KASP markers by us and more added markers may be the best choice for identifying varieties including conventional and hybrid rice, especially in China.

### The Lower Identifying Rate of Conventional *Japonica* May be due to the Narrow Genetic Base

In previous study, the researchers found that the temperate *japonica* had lower average gene diversity and PIC value compared with other subpopulations (Garris et al. 2005). Also, the *japonica* subspecies experienced a more severe bottleneck than the *indica* subspecies (Gao and Innan 2008). For the analysis of the chloroplast genome, a strong positive selection may occur in *japonica* (Cheng et al. 2019). These researches all showed that the *japonica* subspecies especially the temperate *japonica* had lower genetic diversity. So, the 48 KASP markers could not identify *japonica* varieties as precisely as *indica* varieties. Additionally, the conventional *japonica* bred varieties almost came from Jiangsu province, China. The local climate and the soil which differed from other regions limited the emergence of diversity in variety. But for *indica*, more genetic diversity was found than *japonica* from our results and previous study (Xu et al. 2012). The more diversity may be related with diverse environments experienced by *indica* (Wang et al. 2018). The higher genetic diversity caused more combinations and identification more precisely in *indica*.

### More KASP Markers Could be Added According to the Identification of Functional Alleles

In our study, more than 15% of varieties could not be separated from the others. So, we added more KASP markers to obtain a finer resolution in conventional *japonica*. The addition of two markers demonstrated that it could increase the identification ratio in our results. In a previous study, KASP markers were used for functional genes, such as *Wx* and *ALK*. With the progress of modern biological science, more genes will be identified by genome-wide association study (GWAS) (Tang et al. 2019), bi-parental QTL (Wei et al. 2010), or map-based cloning (Xu et al. 2019); thus, more functional alleles will be discovered among different subpopulations, and functional alleles could be used for KASP marker development. More KASP markers combined with the phenotype of varieties will distinguish more accurately, especially in *japonica*. In the subpopulation of Korean *japonica*, 400 markers were suitable for genetic analysis (Cheon et al. 2018). Thus, the identifying rate would meet the requirements of researchers with adding more KASP markers.

### Specific Markers Could be Used for Molecular Identification and Breeding

With the shortage of a labor force and the development of technology, variety identification will enter the era of molecular identification (Pourabed et al. 2015). In our results, the markers with different variations between *japonica* and *indica* may be used for variety identification and then combined with the DUS test to make variety identification more accurately. The heterozygosity of different chromosomes identified in hybrids will provide a reference for cross breeding and suggestions for breeders to select parents. In *japonica* hybrid rice, three important markers could be used as breeding target markers to improve the efficiency of breeding.

## Conclusions

In summary, we selected 48 markers from 378 KASP markers to distinguish different varieties, including conventional and hybrid varieties, and the resolution was about 85%. Through the analysis of the detection rate and heterozygous rate of each variety, we found that more markers could benefit the detection rate. The 48 KASP markers could differ from each other in *indica* and hybrid *japonica*. In conventional *japonica*, the 48 KASP markers could almost identify the varieties from different breeding institutes. The KASP markers in three chromosomes (3, 6 and 11) may have contributed to heterosis according to the heterozygous rate. For KASP marker analysis, three markers were vitally important for *japonica* hybrids.

Overall, we found that less KASP markers could be effective in identifying varieties and assisting rice breeding. The breeders could use the 48 KASP markers to genotype the varieties and add the data to the panel. According to the SNP combinations, the breeders could observe the SNP combination was the same with the panel or not.

## Materials and Methods

### Plant materials

The seeds of 46 and 518 accessions were supplied by Institute of Germplasm Resources and Biotechnology, Jiangsu Academy of Agricultural Sciences, China. The name of all accessions was listed in the Additional file tables. Genomic DNA was extracted from 2-weeks leaf tissues of the rice accessions using the DNeasy Plant Mini Kit (QIAGEN, Hilden, Germany).

### Primer Selection

The 378 KASP markers were selected from the LGC Genomics database. All primers including the name, position and sequence were listed in the Additional file 1: Table S1. After detection the panel with 378 KASP markers, we selected the makers with the detection ratio (> 0.8), MAF (> 0.2), PIC (> 0.3) and physical position. At last, we selected 48 markers that were almost evenly distributed on 12 chromosomes for analysis the 518 varieties. The location of markers was visualized using MG2C tools (Chao et al. 2021).

### KASP Marker Assay

KASP amplifications and allelic discriminations were performed using a Nexar system (LGC Douglas Scientific, Alexandria, USA) in the Jiangsu Academy of Agricultural Sciences. KASP assays were performed using 0.07 µL of 2 × Master Mix and 2.5 µL of 2 × KASP assay mix (LGC Genomics, London, UK) with 2.43 ng genomic DNA in a final reaction volume of 5 µL in a 384-well Array Tape. NTCs (Non-template controls) were included in each run. KASP amplification was performed using the following thermal cycling profile: 94 °C, 15 min; The second step: 94 °C, 20 s, 61 °C, 1 min, 10 cycles in total; Step 3: 94 °C, 20 s, 55 °C, 1 min, 26 cycles in total 94 °C, 15 min; The second step: 94 °C, 20 s, 61 °C, 1 min, 10 cycles in total; Step 3: 94 °C, 20 s, 55 °C, 1 min, 26 cycles in total. The fluorescence measurement was taken for KASP genotyping after PCR amplification. Genotypes of each sample were called using Intellics software (LGC Douglas Scientific, Alexandria, USA). Markers showing clear allelic discrimination were regarded as polymorphic.

### Population Structure Analysis

After integrating all the sites information, we convert the data form into HapMap format. The Admixture software was used for analyze the K. Neighbor-Joining method was used to build phylogenetic tree using Tassel 5 (Glaubitz et al. 2014). The iTOL website (https://itol.embl.de/) was for phylogenetic tree figure. The PCA was analyzed using the TASSEL 5, and the results were visualized using R (https://www.r-project.org).

### PIC, MAF, Detection Rate, Heterozygous Rate and Identifying Rate Calculation

PIC_Calc software was used to calculate the PIC according to the following formula:

$$PIC = 1 - \sum\limits_{j = 1}^{n} {P_{ij}^{2} }$$

The number of detected SNPs and heterozygous sites in the population was observed using TASSEL5. MAF wea calculated using TASSEL5. The detection rate was calculated according to the following formula: Detection rate = detected site (the sites with well-defined genotypes)/all sites.

In hybrid, the heterozygosity of different individuals and sites was summarized using the Tassel 5 and calculated using the following formula: Heterozygous rate = heterozygous sites/all detected sites. The SNP combination was counted of each variety. Secondly, the varieties which shared with the same combination were counted. At last, the ratio of the varieties which has unique combination to the all varieties was the identifying rate.

## Supplementary Information

**Additional file 1: Table S1** The list of 378 KASP markers.

**Additional file 2: Table S2** The list of 46 varieties.

**Additional file 3: Table S3** The 331 KASP genotype of 46 varieties.

**Additional file 4: Table S4** The list of 50 KASP markers.

**Additional file 5: Table S5** The result of CV error produced by Admixture.

**Additional file 6: Table S6** The result of PCA of 46 varieties using 331 markers and 48 markers.

**Additional file 7: Table S7** The list of 518 varieties.

**Additional file 8: Table S8** The 48 KASP genotype of 325 conventional varieties.

**Additional file 9: Table S9** The 48 KASP genotype of 193 hybrid varieties.

**Additional file 10: Table S10** The list of conventional japonica after deleting the possible redundant sib lines from the same breeding institutes.

**Additional file 11: Table S11** The result of CV error produced by Admixture.

**Additional file 12: Table S12** The result of PCA of 325 conventional varieties using 48 KASP markers.

**Additional file 13: Table S13** The reference allele frequency.

**Additional file 14: Table S14** The result of Loglikelihood (LK) produced by Admixture.

**Additional file 15: Table S15** The result of PCA of 193 hybrid varieties using 48 KASP markers.

## Data Availability

The datasets supporting the conclusions of this article are included within the article and its additional files.

## Abbreviations

KASP: Kompetitive allele-specific PCR
DUS: Distinctness, uniformity, and stability
SNP: Single nucleotide polymorphism
GBS: Genotyping by sequencing
HRM: High resolution melting
SSR: Simple satellite repeat
InDels: Insertion/deletions
QTL: Quantitative trait loci
MAS: Marker-assisted selection
PIC: Polymorphism information content
MAF: Minor allele frequency
GWAS: Genome-wide association study
